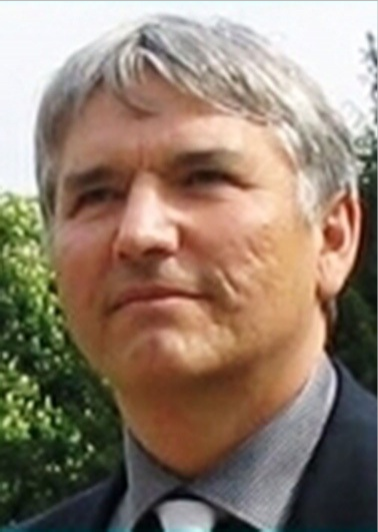# Caveat Usor: Assessing Differences between Major Chemistry Databases

**DOI:** 10.1002/cmdc.201700724

**Published:** 2018-02-23

**Authors:** Christopher Southan

**Affiliations:** ^1^ IUPHAR/BPS Guide to PHARMACOLOGY, Deanery of Biomedical Sciences University of Edinburgh Edinburgh EH8 9XD UK

**Keywords:** bioassays, chemistry, databases, patents, structures

## Abstract

The three databases of PubChem, ChemSpider, and UniChem capture the majority of open chemical structure records with February 2018 totals of 95, 63, and 154 million, respectively. Collectively, they constitute a massively enabling resource for cheminformatics, chemical biology, and drug discovery. As meta‐portals, they subsume and link out to the major proportion of public bioactivity data extracted from the literature and screening center assay results. Therefore, they not only present three different entry points, but the many subsumed independent resources present a fourth entry point in the form of standalone databases. Because this creates a complex picture it is important for users to have at least some appreciation of differential content to enable utility judgments for the tasks at hand. This turns out to be challenging. By comparing the three resources in detail, this review assesses their differences, some of which are not obvious. This includes the fact that coverage is significantly different between the 587, 282, and 38 contributing sources, respectively. This not only presents the “who‐has‐what” question, but also the reason “why” any particular inclusion is considered valuable is rarely made explicit. Also confusing is that sources nominally in common (i.e., having the same submitter name) can have significantly different structure counts, not only in each of the three but also from their standalone instantiations. Assessing a series of examples indicates that differences in loading dates and structural standardization are the main causes of this inter‐portal discordance.

## 1 Introduction

### 1.1 Overview

This trio of PubChem (PC), UniChem (UC), and ChemSpider (CS) are the largest public data sources for bioactive chemistry and drug discovery.^1^, ^2^, ^3^ Crucially, their funding has allowed each of them to maintain a steady rate of content expansion from the subsumation of new sources. This review cannot cover all the features of this impressive triad, but the focus will be on providing insight into differential content, the complexities thereof, and how this translates to complementarity. Because content is the principal value proposition for any database, it is important for users to appreciate differences when deciding relative utility. In addition, knowledge of the contributing sources can indicate where there are advantages to query these directly, via their standalone instantiations, rather than what may be stripped‐down records subsumed into integrated resources.

It is assumed that readers of this article are not only aware of PC and CS but have had at least some experience using one or the other. The community familiarity with the youngest of the three, UC, is likely to be lower (although ChEMBL and SureChEMBL users may have noticed the UC cross‐references nested in each compound record). It should also be noted that most researchers assessing the use of integrated sources would include SciFinder (SF) as the fourth major source.^4^ This currently declares 134 million organic and inorganic chemical substances (although at 105 million, the Reaxys commercial offering is not that far behind). Via academic departments and pharmaceutical company licensing, many cheminformaticians are thus likely to have access to the “big four”.

This review focuses on the three public resources, as their details are largely open. However, the approaches outlined for content dissection and comparison (a.k.a. slicing and dicing) can also be applied to commercial databases (depending, of course, on what their internal query functionality allows). In context, it is important to note that, despite the adjective of “proprietary” being often applied to describe licensed databases, their content is drawn entirely from public primary sources. Notwithstanding, selective capture means they may still contain a proportion of unique structures.^5^ The challenges associated with divergent expansion of commercial and public sources were reviewed in 2015 (this includes discussions and references to quality aspects that cannot be covered here).^6^ However, this new analysis includes more comparative detail and covers recent changes in the public “big three” (n.b., all numbers reported herein were initially harvested in November 2017 with some post‐refereeing updates in January 2018).

## 2 PubChem

### 2.1 Growth

**Figure 1:**
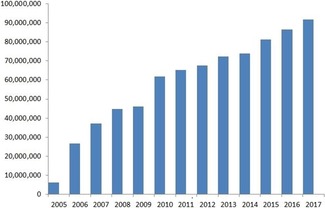
Cumulative PubChem content by year. *Y*‐axis values represent the numbers of Compound Identifiers (CIDs).

Despite being in the middle in terms of size ranking, we can begin with the description of PC as an archetype against which to compare the other two databases. Growth since 2005 shows an approximately linear increase in just over a decade, now approaching 95 million distinct chemical structures (Figure 1).

Those cheminformatics practitioners who remember LBC (life‐before‐PubChem) may be more appreciative than their younger colleagues of what an achievement this represents for an open public resource, funded by the US National Institutes of Health (NIH). The obvious feature is that the increase is approximately linear, in contrast to the exponential growth of sequence data for the sister discipline of bioinformatics. So why is this? The minimum parsimonious assumption is that global output over this period was related to the number of chemists making compounds. The chart of Figure 1 indicates that long‐term growth has been sustained (although there has been distinct slowdown during 2017), despite the shrinking of medicinal chemistry resources in the pharmaceutical sector over the same decade (i.e., fewer people in companies making compounds).^7^ Concomitantly, there is no clear sign of automated chemical synthesis accelerating output (but this could change). While the subject will be expanded on later, a proportion of this total is certainly derived from enumerated virtual structures that have never been made. Notwithstanding, there is no evidence these are making a significant contribution to the overall growth rate.

**Figure 2:**
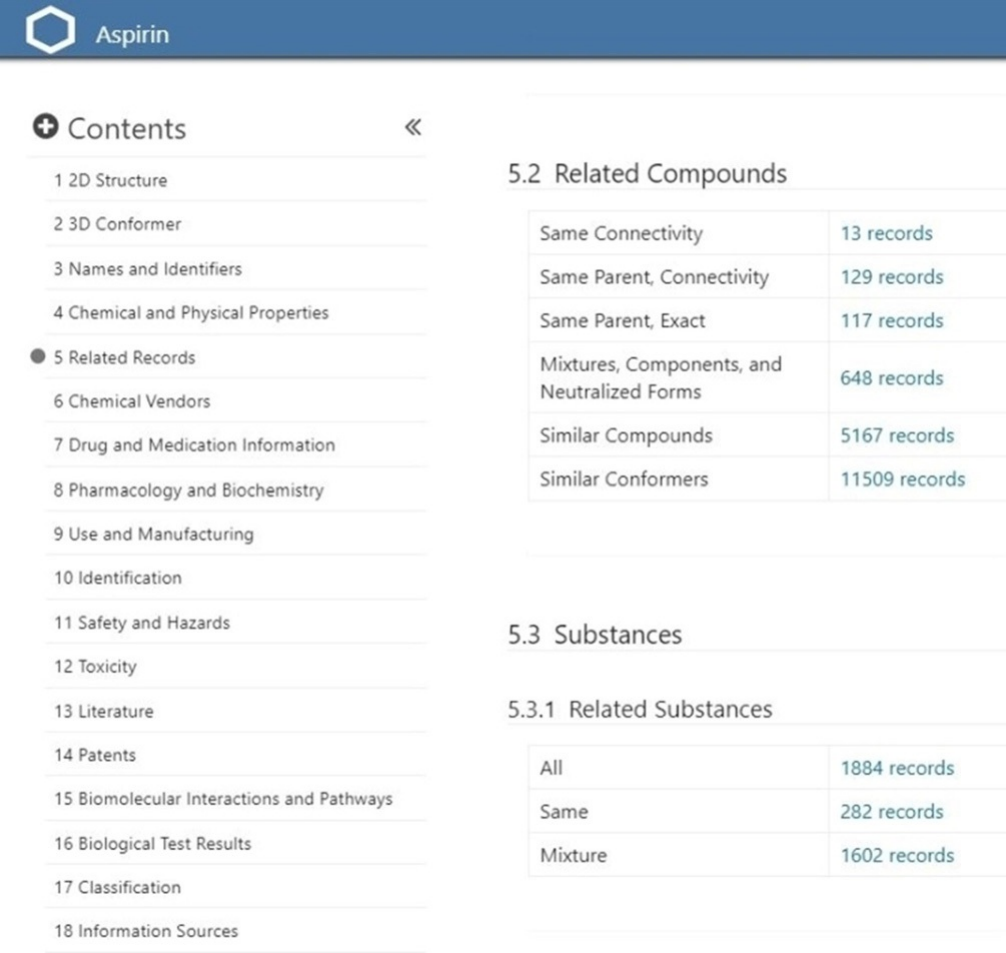
Snapshot of the related CID and SID records for aspirin in PC (https://pubchem.ncbi.nlm.nih.gov/compound/2244, InChIKey BSYNRYMUTXBXSQ‐UHFFFAOYSA‐N) on the right and the structural relationship indexing on the left.

As for all three databases, PC is submitter‐based. This means that chemical structures conforming to standardization rules are accepted as primary database records assigned to each discrete submitter by means of Substance Identifiers (SIDs). These are then merged, according to PubChem chemistry rules, into non‐redundant Chemical Identifiers (CIDs). Consequently, the 236 million SIDs, from different PC submitters, merge to 94 million CIDs, representing an average of just over 2.5 SIDs per CID. However, this is a heavily skewed distribution because 48 million CIDs in PC are unique as defined by being derived from a single source (i.e., have only one SID). As we can see from a breakdown of the top‐ten submitters (Table 1) these already cover substantial amounts of single‐source structures. These contrast with more “popular” subsets, for example, approved drugs, where this is reflected in having many submitters. Using a selection query from the SID side we can establish that the source IUPHAR/BPS Guide to PHARMACOLOGY (GtoPdb) includes 1180 approved drugs as CIDs (as of release 2017.5).^8^ Transforming these across to their contributing SIDs indicates a CID/SID ratio of 1:110. However, this average also covers a skewed distribution where newer approved drugs generally have fewer submitters than old drugs. We can thus take aspirin as one of the most “popular” examples to discern the extent of multiplexing (the same structure in different sources). The PC source mappings are indicated in Figure 2.

**Table 1:** Largest submitting sources in PubChem selected at 92 058 388 CIDs and 230 507 344 SIDs.

Source	Count^[a]^	Unique^[b]^	Last update
1. Aurora (v)	34.3	13.0	2016/02/15
2. AKos (v)	32.2	8.8	2017/09/19
3. SureChEMBL	17.4	8.3	2017/08/04
4. IBM	15.2	2.7	2017/01/26
5. ChemSpider	14.6	1.1	2011/10/24
6. ZINC (v)	13.8	7.0	2017/08/26
7. DiscoveryGate	11.4	0.70	2011/05/05
8. NextBio	10.2	0.02	2009/06/13
9. ABI Chem* (v)	7.9	0.07	2011/04/01
10. MolPort (v)	6.6	0.06	2017/06/19

[a] Counts refer to SIDs in millions. [b] Unique content is counted as single‐source CIDs, also in millions. ✶: Indicates a legacy submission. (v): Aggregators of vendor catalogues. Data were extracted from https://pubchem.ncbi.nlm.nih.gov/sources/, which includes links for each individual source.

Thus, acetylsalicylic acid (aspirin) has 282 single structures, plus 1602 mixtures, as SIDs. But these collapse to 648 distinct CIDs, inferring that some different sources are submitting identical mixtures. The “same connectivity records” (as distinct CIDs) turn out to be 13 isotopically labeled derivatives.

### 2.2 PubChem major sources

PC has 548 data sources, but only 488 of these have live (i.e., not on‐hold) substance counts. This is because some contribute annotation links but not structures (e.g., ClinicalTrials.gov contributes 7788 annotations). The distribution has a long tail, where 282 have more than 1000 SIDs but over 90 sources have 10 or fewer SIDs (including the author's own submitted set of 10 as “TW2Informatics”[SourceName]). The top ten are listed in Table 1.

We will return to the Table 1 features when comparing the other sources, but some aspects can immediately be picked out. The first is the dominance of vendor aggregators. Another feature is that automated patent extraction comes in as second (sources 3 and 4).^9^ However, 5, 7, and 8 are not straightforward to classify; indeed 7 and 8 have neither source outlinks nor metadata in the SIDs. Thus, along with 9, these are arguably legacy submissions, as neither has updated in recent years. Uniqueness is a key aspect of databases, but it is not always clear what this means for users. The numbers in Table 1 are defined by the chemistry rules for the formation of CIDs, although the details of these are not completely exposed in PC. However, the relationship computation and query navigation allows a level of exploration to divine what these rules actually are by observing the consequences they have in mapping results. Arguably, these can be considered stringent in the sense that differences in isotopes, stereochemistry, tautomers and mixtures all lead to the formation of distinct CIDs and InChIKeys (i.e., a philosophy of splitting rather than merging).

### 2.3 PubChem features

**Figure 3:**
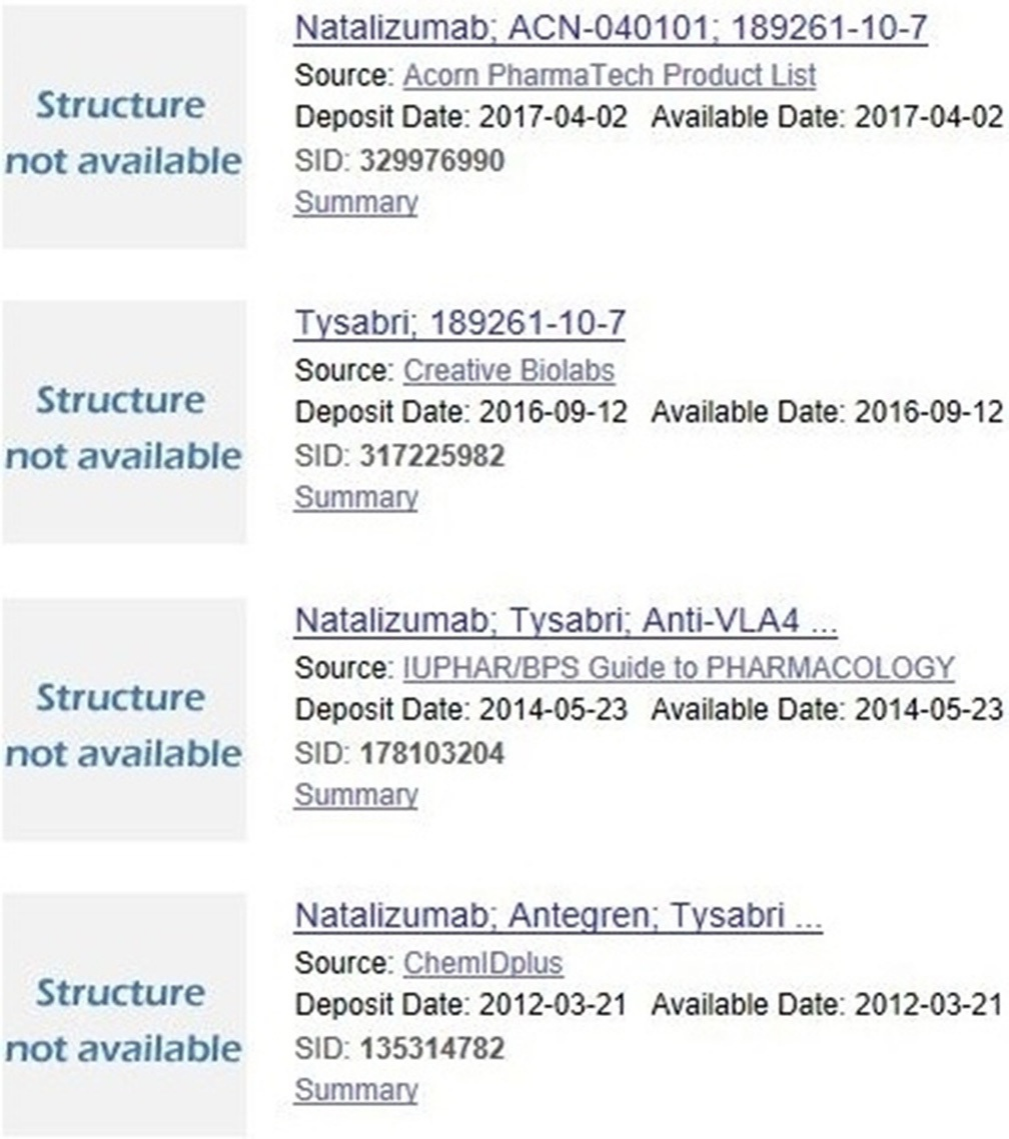
Selected SIDs retrieved with the approved clinical monoclonal antibody natalizumab. “Structure not available” indicates that neither a CID could be formed nor a rendered chemistry image. Note the spread of submission dates from these particular sources over four years.

The other two databases solve the necessary redundancy collapse of submissions in a similar way to the SID/CID splits, but where the InChI system plays a more central role.^10^ However, there are unique aspects of PC that should be mentioned. The first of these is that the substance submissions include entries that cannot form CIDs, since they have not been transformed into SMILES or SD files by the submitters. To become a CID, the SIDs have to be within the current upper limit of 1000 atoms, approximating to ≈70 residues for a peptide. Those excluded from CID merging are thus larger peptides, polynucleotides, or siRNA reagents but also include biological therapeutics such as antibodies that have a designated INN or a clinical candidate designation. For example, a substance search with the INN “natalizumab” includes the SIDs shown in Figure 3.

Thus PC indexes biologics of various kinds from different sources but cannot merge them. Because in UC and SC everything has to conform to chemistry rules, they do not currently have this category of submission. The second key difference is the surfacing of biological activity data in PubChem BioAssay (this can also include SIDs without CIDs). The many aspects of this third dimension of PC data cannot be detailed here, but they are crucial for PC′s value to chemical biology and drug discovery.^11^ The top‐level statistics are that 2.4 million CIDs have been tested in 1.2 million assays (i.e., with distinct assay identifiers as AIDs) and 1.1 million of these have at least one SID recorded as “active”. Compared to a typical HTS assay, where hit rates are usually threshold filtered to around 1 %, there are clearly caveats with the definition of “active” when this is as high as 45 %. However, given that ChEMBL submissions dominate BioAssay (with 1.24 million entries compared with only ≈1000 each for the next four screening centers, ranked by AID counts) there is a clear bias toward positive results extracted from 67 722 papers into ChEMBL 23 (but note these include inactives from the same papers). BioAssay contains a small proportion of alternative uses of this as a submission category. For example, the 1216 SIDs in AID 1195 are approved drugs with US Food and Drug Administration (FDA) Maximum Daily Dose assignments (i.e., not assay results). BioAssay also has what appear to be false negatives in the information capture sense. For example, a simple INN query (which, from a limited number of record checks, seemed to be substantially true positives) retrieves 8949 CIDs, but only 4399 of these have BioAssays scored as “active”. Because INNs are specific for advanced development compounds (mostly reaching at least Phase 1 clinical trials) accrued over many decades, we would expect the majority to have published bioactivity. We can test possible explanations directly from the PC source intersects. For example, only 3926 are in the NIH Molecular Libraries screening collection (and these have not all been screened against probable molecular targets anyway). We can also determine that ChEMBL has captured 7053 of the same structures but only recoded “actives” for 4321 of those. Thus, nearly half the INNs have neither explicit activity data against human protein targets reported in publications that ChEMBL has extracted, nor have accumulated such data from the screening centers. Looking at examples indicated at least three causes: firstly new INNs, secondly old INNs, and thirdly the BioAssay data had been assigned to a different stereoisomer. An analogous inference of significant false negatives also applies to the 15 514 CIDs that Medical Subject Headings (MeSH) annotators have classified as having pharmacological action by curation from PubMed. While these are implied to be active in vivo, only 7016 have positive BioAssay results.

Another key difference is the integration of PC into Network Entrez.^12^ This is a powerful and extensive system for the cross‐referencing of information about biological and chemical entities from the 136 databases within the NCBI. This includes the direct connectivity between Compound, Substance, BioAssay, protein sequences, protein structures, and BioSystems (pathways). Another feature that presents an advantage for PC is the ability to upload, immediately visualize and then download, Entrez result sets from bulk queries. This can be accomplished either via the Structure Search file upload or the PubChem Identifier Mapping Service web page (capacity may vary, but can be in the thousands). For inter‐database comparisons this means smaller sources from other databases with appropriate download options (e.g., SDF, SMILES, and InChI) can be mapped “into” PC to determine exact intersects and differences.

## 3 UniChem

### 3.1 UniChem features

UC is very different from PC in being a large‐scale database of pointers between chemical structures. This means, unlike PC and CS, it does not store the actual structures (e.g., as SD files or SMILES), but these can be accessed via the source URLs. Initially conceived to integrate chemistry across the internal EBI databases (ChEBI, ChEMBL, SureChEMBL, PDBe, and most recently Metabolites) it now extends to 32 external sources. It is also designed to enable “on‐the‐fly” linking via REST web services. It is also designated with a CC‐0 license as specified on the website. The different automated downloading procedures and loading dates are summarized for each of the 37 sources: These are compiled into the weekly release, regardless of individual source update cycles. The InChIKey (IK) centric cross‐pointing and source redundancy reduction is conceptually similar to the SID merging rules in PC. It also uses features of the Standard InChI to enable mappings between molecules that share common atom connectivity via the inner Key layer. Importantly, this extends across isotopes, stereo forms, mixtures, and salts. This is analogous (but again, not exactly equivalent) to the PC “same connectivity” relationships between CIDs identifier mapping service.

### 3.2 UniChem major sources

**Figure 4:**
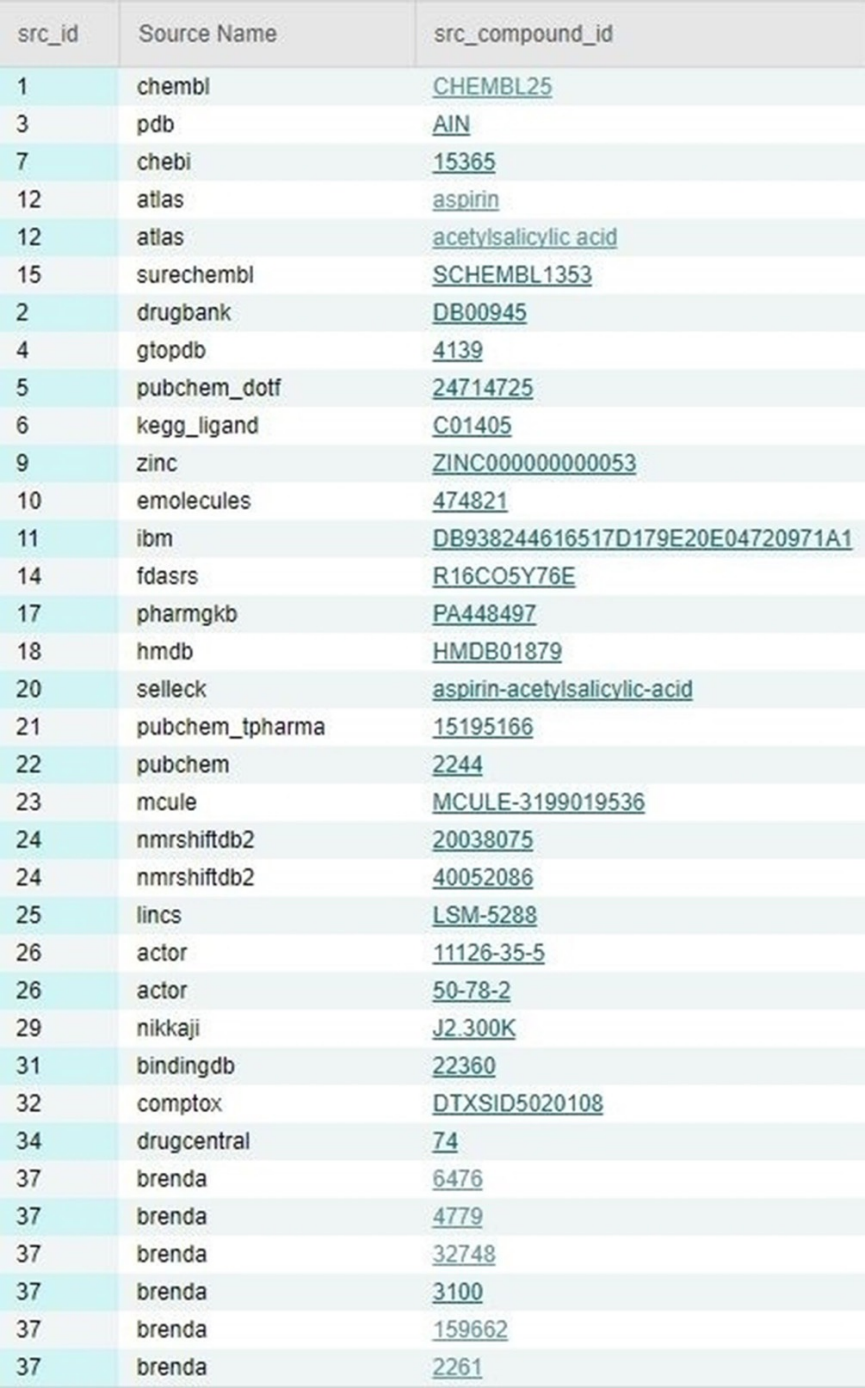
The UC source listings associated with the full InChIKey for aspirin BSYNRYMUTXBXSQ‐UHFFFAOYSA‐N (lower‐case abbreviations are corrected where referred to in the text).

The top‐ten source counts are shown in Table 2. We can return to Table 2 when discussing comparative content, but some unique characteristics of UC can be introduced. As an approximate (but not exact) equivalence to PC CID:SID ratios, the UC structure:assignment ratio is 1.37. This is not unexpected, as there are fewer sources. Unsurprisingly, PubChem becomes the major source contributor, corresponding to 30 % of all unique structures. However, as UC point out, because many sources are loaded into both PC and UC independently (i.e., twice) this confounds the intra‐database statistics. Comparative insights can be gleaned from the entry for aspirin in Figure 4.

**Table 2:** Largest sources in UniChem selected at 151 231 603 structures and 207 117 159 assignments, release 153, 2017/09/10.

Source	Count^[a]^	Unique^[b]^	Last update^[c]^
1. PubChem	91.5	35.8	2017/09/06
2. Mcule (v)	32.9	23.8	2016/02/29
3. Molport (v)	22.4	0.14	2014/12/08
4. SureChEMBL	18.6	2.2	2017/09/05
5. ZINC (v)	16.9	1.1	2017/08/21
6. IBM	7.9	0.31	2015/02/11
7. Emolecules (v)	5.2	0.07	2012/03/15
8. tpharma	3.8	0.1	2009/06/13
9. nikkaji	3.2	0.3	2011/04/01
10. ChEMBL	1.7	0.06	2017/05/24

[a] Source counts for src_id refer to full InChIKeys in millions. [b] Unique content for that source are also full InChIKeys. [c] Last update date is taken from the source descriptions (not the release date). (v): Vendor. Data were extracted from https://www.ebi.ac.uk/unichem/analysis/unique, where source links are included.

**Figure 5:**
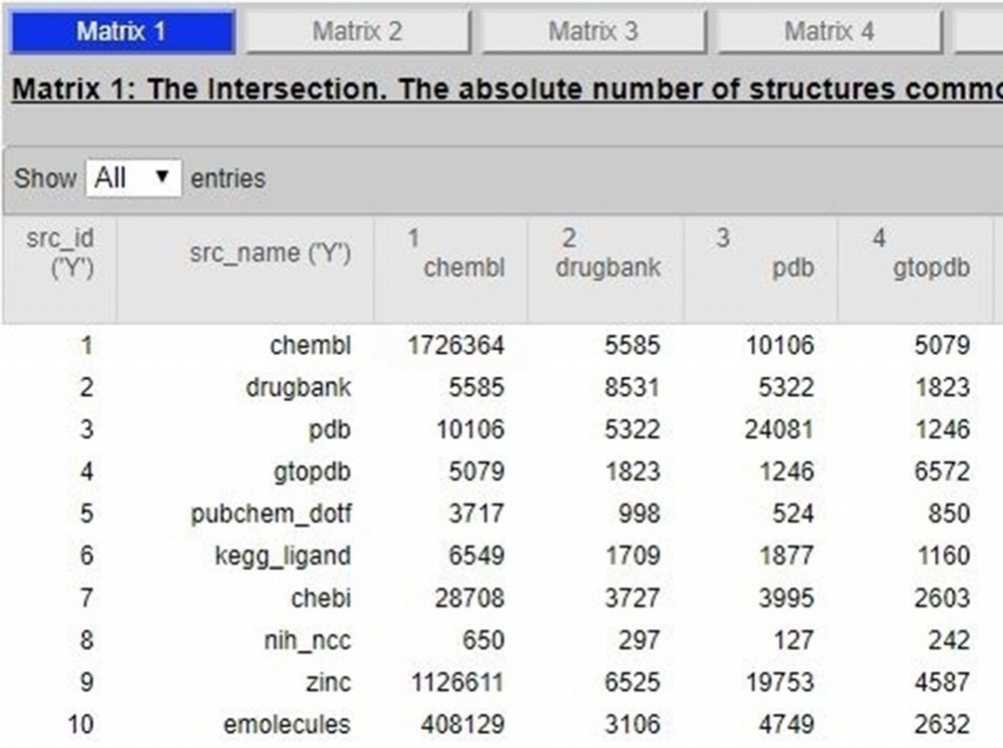
A snapshot from a section of the source comparison matrix.

We can see that, as IK matches, aspirin is indexed in 27 of the 37 sources. In addition, there are some multiple records on the source side for identical structures (e.g., Atlas, NMRShift, ACToR, and BRENDA). Another unique aspect of UC is the computation of different forms of equivalence between sources, as no less than seven 37×37 overlap matrices at different comparison stringencies. This is explained in detail on the site and the publications. A small section of the results based on the full IK is shown in Figure 5.

The matrix can be read as follows for GtoPdb (the IUPHAR/BPS Guide to PHARMACOLOGY): The total (i.e., row and column four) is 6575. The overlap with ChEMBL (as IKs in common) is 5079, DrugBank 1823, and PDB (i.e., the heteroatom small‐molecule entries) is 1246.

## 4 ChemSpider

### 4.1 Basic features

**Figure 6:**
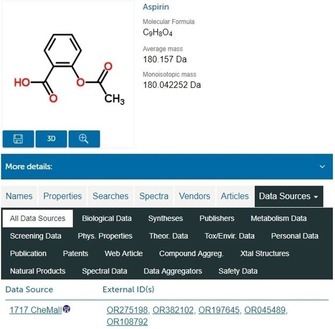
The ChemSpider entry for aspirin http://www.chemspider.com/Chemical-Structure.2157.html.

CS is owned by the Royal Society of Chemistry (RSC). While searches and extraction of limited result sets are free, it is not open data, so download of the entire database is only available under license. One of the important consequences of this is that it is not subsumed into UC (as is PC). It offers services to improve submitted data by user corrections (e.g., of names to structures), added annotations, and integration with user applications. This extends to ChemSpider SyntheticPages, which covers reactions with citable URLs, peer review, and semantic enhancement. Another unique feature is direct links to structures in RSC journals. Like the other two databases it has merging rules for structure and records point to multiple submitting sources. The CS example for aspirin is shown in Figure 6.

By executing a search with the InChIKey inner layer (also sometimes termed a skeleton match), 160 data sources are indicated, but some of these are multiplexed by one‐to‐many entries. For example, the 10 ChemIDplus entries include nine mixtures and the nine Crystallography Open Database entries are each from distinct 3D structure determination references. While this multiplexing would expand to well over 160 individual links, these are still substantially less than the analogous 1884 PC SID entries in Figure 2. As outlined below, both CS and PC contain a similar number of large sources, indicating that the disparity is due to organizational and chemistry rule differences between the two. These are complex in both cases arising from the challenges of integrating many different sources.

### 4.2 ChemSpider major sources

The top‐ten sources for CS are listed in Table 3. Compared with PC and UC there is a notable absence of the large automated patent sources of SureChEMBL and IBM (Discovery Gate did have a feed from Derwent manual patent extractions, but this source is no longer active). Comparing Tables 2 and 3 indicates a reciprocal cross‐pointing anomaly, where CS points to 12.9 million PC entries but only pre‐2015. PC points to 14.6 million CS entries, but these are pre‐2011 (the last‐update dates represent revocations, not structure change, as the bulk of the reciprocal cross‐referencing was added between 2007 and 2008). Because both databases have expanded extensively in the intervening years, as has been pointed out, the value of such legacy partial cross‐pointing is questionable.^13^

**Table 3:** Largest sources in ChemSpider (November 2017).

Source	Count^[a]^	Last update^[b]^
1. Aurora (v)	25.2	2016/06/12
2. PubChem	12.9	2015/06/25
3. AKos (v)	10.4	2017/09/26
4. Mcule (v)	5.6	2010/10/30
5. Molport (v)	5.3	2014/09/02
6. eMolecules (v)	4.8	2009/06/08
7. ZINC (v)	3.8	n/a
8. Otava Chemicals (v)	3.2	2017/09/22
9. LabNetwork (v)	2.8	2017/10/11
10. Discovery Gate	2.5	n/a

[a] Counts refer to compounds in millions. [b] n/a: Last update date was not available on the website. (v): Vendor aggregator. Data were extracted from http://www.chemspider.com/DataSources.aspx.

## 5 Comparative Content

### 5.1 Sources in common

**Figure 7:**
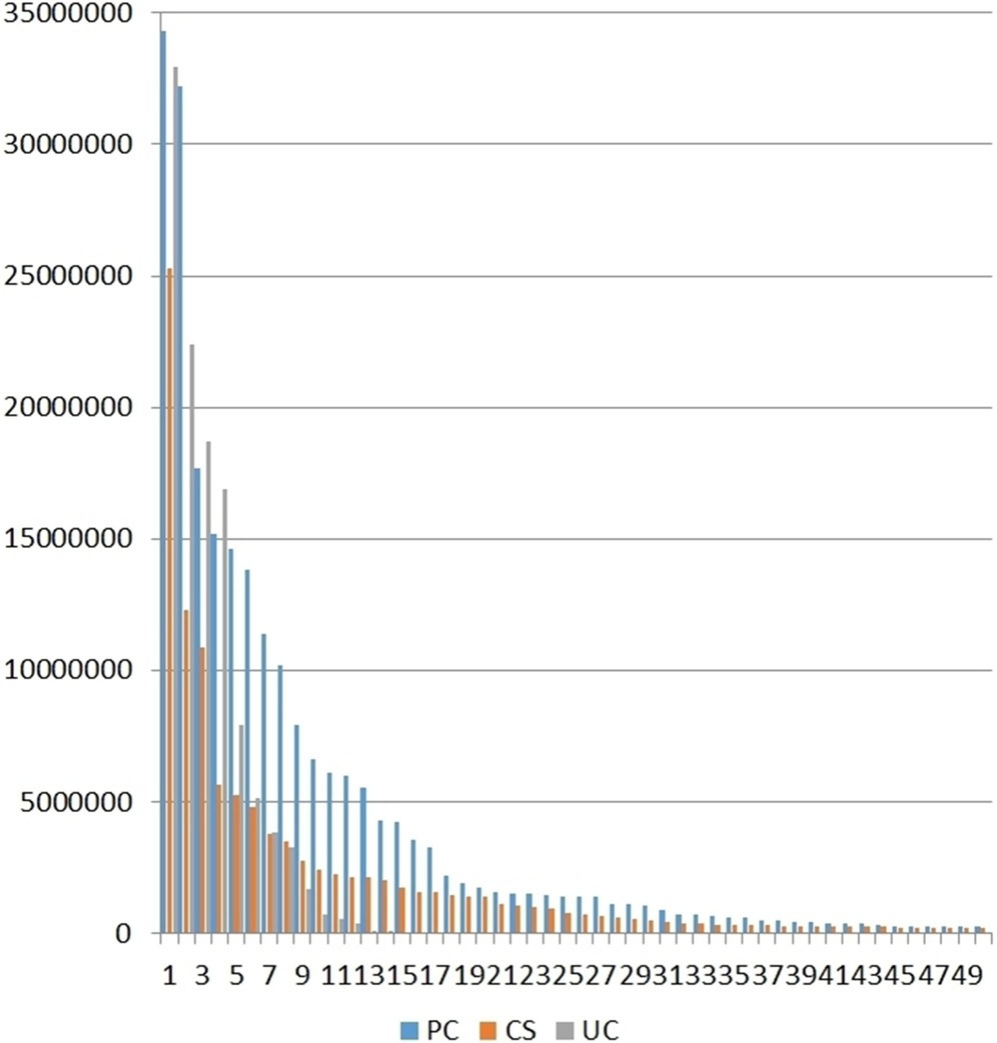
Distribution of source sizes in PC (blue) CS (red) and UC (grey). These are plotted for the top‐50 of PC and CS, where the cutoffs were 280 000 and 207 000 respectively. For UC, 36 of the 37 sources are plotted with the exception of PC (within UC) that would be off scale at 92 million.

**Figure 8:**
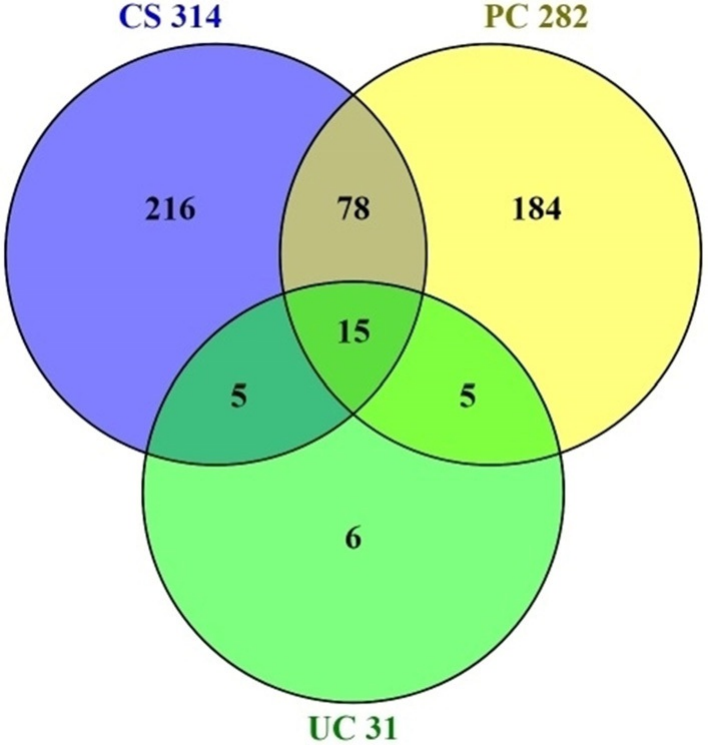
Normalized name intersects for sources with greater than 1000 structures between the three databases. Total source counts are given next to each name.

Differences between the major sources are listed in Tables 1–3. However, we can take a more detailed look at the distributions of the top 50 (Figure 7). As expected, because it contains only 34 sources, Figure 7 shows a steep fall‐off in UC. While the other two both show a long tail, it is clear that CS is dominated by more smaller sources than PC. We could get more insight by comparing these by name. However, it is already clear from Tables 1–3 that some names are different but probably related. For example, “Aurora Fine Chemicals LLC” in PC corresponds to “Aurora Feinchemie” in CS (i.e., both the US and German links connect through to the same website, but UC does not include their feed). Similarly, “Thomson Pharma” has the same name in both CS and PC, but in UC it is named “pubchem_tpharma”. Yet another example was the pointers to different Singapore or Chinese web addresses for Angene in CS and PC. By standardizing names, at least for the larger sources, it was possible to get an outline of divergence. The result is shown in Figure 8.

We can see from Figure 8 divergence between PC and CS but some degree of convergence with UC in that over 70 % of its sources are shared with either of the other two. We can pick out a selection of unique sources to get an idea of differential value (even if there are overlaps between most sources). Comparing Figures 7 and 8 indicates that CS has on the order of 200 unique smaller sources, at least as judged by having a different name (n.b., it was not feasible to check all the websites to rule out if some had the same origins). One challenge here is discrimination of primary vendors versus aggregators. The former, by implication, are assumed be the primary manufacturers of the compounds or at least holding them as local stock. The majority of the “long‐tail” sources in CS and PC are probably in this category. Aggregator (or secondary) vendors are brokers of many merged primary vendors and thus appear as some of the largest sources in the top‐ten of all three databases. While some may self‐declare as one or the other, without effecting some kind of due diligence it is difficult to cleanly discriminate secondary from primary (so use of the term “vendor” from this point on will not be qualified with such a distinction).

### 5.2 Unique sources

This refers to sources that appear in only one of the three databases (but note, this does not imply unique content). For CS the largest unique vendors, ranking at eighth and ninth, are Otava Chemicals and LabNetwork with 3.5 and 2.8 million compounds, respectively. Sources of more chemical and biological interest include: Journal of Heterocyclic Chemistry 184579, FooDB with 16744 food ingredient compounds, Royal Society of Chemistry (abstracts) 149831, The National Compound Collection with 42 779 structures from UK Chemistry PhD theses, StreptomeDB with 3780 *Streptomyces* metabolites, and the Toxin‐Target Database with 3192 entries. Another interesting aspect of CS is that three named individuals exceed the 1000 cutoff, with SE at 7033 and two CS team members, DS with 2244 and SR with 1055.

As we can see from Figure 8 PC also has many unique sources (while one of these is PubChem BioAssay, this can be classified as a collection of sub‐sources), but space limitations preclude more than a few to be noted here. It is useful to be able to read off the explicit uniqueness within PC internally by simply adding “1[DepositorCount]” to source selects. For example, the automated patent extraction source SCRIPDB has 4.0 million CIDs, of which 0.48 million are only from that source. In the case of Collaborative Drug Discovery, 0.87 million of their 1.40 million are unique. As one of the smaller sources, WikiPathways indexes 1997 structures with only 23 unique. Some larger PC‐only sources have low levels of uniqueness, as we can see for the figure of 0.2 % within the 10.2 million from NextBio. These turn out to be re‐submission errors that have connectivity to other existing CIDs, confirming that this source has simply performed a one‐off extraction and resubmission of pre‐existing content labelled as their own SIDs in June of 2009.

One recent new source (October 2017) is from Springer Nature for their journal connectivity initiative.^14^ This is currently at 0.61 million CIDs, of which 0.25 million are unique. A crucial advantage for internal comparisons of sources within PC is the facility to perform Boolean intersects between queries. This can not only reveal the exact overlap and difference between sources A and B, but can be extended to many combinations and the use of filters (e.g., mixtures can be counted as two or more noncovalent units).

From the data in Figure 8, we can ascertain that UC has six large sources not in CS or PC. Intra‐database overlaps between sources can be calculated in a variety of ways depending on the definition of structural identity. As explained in the UC documentation this is reported at three levels, as: 1) identity of the full IK, 2) the connectivity layer of the IK, and 3) the connectivity layers of multiple molecular components (e.g., salt splitting). However, it should be noted that intra‐source comparisons within UC will reflect circularity from the co‐integration of PC into UC. The largest unique source is the US Environmental Protection Agency (EPA)′s Aggregated Computational Toxicology Online Resource (ACToR) at 411229 (4.7 % unique). This is followed by the BRENDA Enzyme information system with 119395 (37 % unique), Lincs (Library of Integrated Network‐based Cellular Signatures) 41802 (0.2 % unique), MetaboLights (sic) 19789 (no unique structures), PharmGKB (Pharmacogenomics Knowledgebase) 1633 (no unique structures), and the Recon knowledge base of human metabolism, 1529 (18 % unique).

### 5.3 Source differentials and dates

This section describes comparing counts for nominally same sources across the databases as well as their standalone instantiations. In some cases, these can have occupancy in all four categories (i.e., three databases and In situ), but we can also consider three‐way and two‐way cases. A selection of these is listed in Table 4.

**Table 4:** Counts for sources with the same name between two or more options.^[a]^

Source	CS	PC	UC	In situ
HMDB	81 950	9947	79 362	114 101
BindingDB	410 612	645 152	538 351	631 854
DrugBank	7082	7399	8684	9041
ChEMBL	1 733 993	1 729 327	1 726 364	1 735 442
Mcule (v)	5 649 580	6 040 174	32 919 693	<35 mill
ChEBI	84 434	90 370	88 199	52 939
EPA	671 300	756 943	719 884	758 000
PDB	24 001	35 457	24 423	25 057
GtoPdb	6632	6822	6572	6702
Thomson Pharma	2 048 770	4 338 420	3 858 588	n/a
MolPort (v)	5 292 001	6 530 473	22 381 196	45 230 189
ZINC (v)	3 769 618	13 751 641	16 886 865	<100 mill
Aurora (v)	25 290 243	33 345 947	n/a	18.1 mill
SureChEMBL	n/a	17 618 128	18 782 006	n/a
FDA/SPL/UNII	61 801	91 364	n/a	93 363
DrugCentral	n/a	4007	3959	4509
IBM	n/a	10 714 534	7 930 403	n/a
eMolecules (v)	4 840 249	n/a	5 168 336	5.9 mill
Carotinoids DB	1067	n/a	1138	1169

[a] The inclusion of n/a means either that particular source was absent, or the local website did not provide an explicit total; these should be distinct compound counts in the three portals, but may be substance records In situ. (v): Vendor. Approximate figures from vendor websites are in millions. Note this matrix was compiled in November 2017 and represents just a selected sampling of differences.

This matrix of discrepancies from nominally identical sources is surprising from a cheminformatics standpoint. Indeed, not even one single pair agree exactly. Relatively minor differences, on the order of a few percent, are not unexpected. These can be attributed to differences in chemistry standardization rules, loading filtration stringencies and, in the UC case, generation of Standard InChIs. However, as can be seen in Table 4, many showed bigger differences in numbers, some of which could be plausibly explained, others would need additional investigation. The most common reason seems to be loading dates. This issue is always problematic for large integration efforts, especially where sources frequently update. This is inherently a good thing, but leaves the meta sources with two challenges: The first is their internal synchronization and submission processing times. The second is the balance between “pulling” and “pushing”. These terms refer to the host portal either actively picking up (pulling) the source data, for example, as ftp and/or an automatic extract, transform and load procedure (ETL), or the submitter sends (pushes) their update manually. In regard to ascertaining dates as possible causes of differences, PC makes these query‐selectable for all submissions (i.e., as SID dates). CS does not surface record dates in the interface, but does indicate first and latest upload dates for most sources. UC operates a weekly rebuild and automatically assigns that date to each source for the computed statistics. However, this is slightly misleading in that it is the source descriptions that include both the first and the latest actual load dates, regardless of the weekly release date.

We can look at selected rows in Table 4 to pick out both concordances and discordances. As an example of the former, we can see a less than 1 % difference between the highest and lowest of the four ChEMBL counts. We can check that ChEMBL 22 from September 2016 In situ updated to version 23 in May of 2017. With this long release cycle the different loads would not affect the October 2017 numbers. We can check this by establishing that the UC load date was the 24th of May (but the data are within the same EBI infrastructure anyway). This was closely followed by CS on the 25th of May and processed within PC on the 6th of June according to the SID dates. In this case, the 6115 difference between PC CIDs and In situ can be explained by those peptide and protein substances that do not have CIDs. While this is supported by the SID count of 1735576, it exceeds the In situ count by 134, but this is a minor discrepancy. Other sources also show small differences such as the IUPHAR/BPS Guide to PHARMACOLOGY. However, this has a relatively rapid release schedule of six per year.

The discrepancies recorded for Thomson Pharma are far from minor, in that the PC count is more than twice that for CS. Establishing the reason for this major difference is confounded by Clavariate (previously Thomson Reuters) having ceased their PC cumulative feed at 4.3 million CIDs in January 2016 (the last SID date) for reasons that remain unclear and not declaring an in situ count. However, inspecting CS source dates indicates the 2.04 million was probably an early load from 2008 (possibly direct). The UC source page records that their set was selected from PC (i.e., as a secondary source) at the end of July 2013. This explains why their 3.8 million lies midway between the CS and PC counts. This means that users wanting to search against this large, high‐quality compilation of manually curated structures from patents and papers would need to query PC for complete (even if now lapsed) coverage.

The ligands in PDB are a crucial small‐molecule set for drug discovery, but come with particular challenges. Firstly, because bioactive chemicals specifically bound in protein pockets are difficult to define and filter cleanly (but are in the order of ≈8000), sources submit all the heteroatom structures (HETATM). These encompass resolved small molecules including salts and reagents. Secondly, there are four different sources within wwPDB and the NCBI. While PDBe and PDBj counts are more or less concordant at 25 057 and 25 252, respectively, RCSB PDB drops slightly to 24 140. It is not clear which of these sets was loaded into CS on the 25th of May, but UC refreshed their internal (EBI) PDBe on the 6th of November 2017. However, we are left with the anomaly of the new PC source of ligand extraction as NCBI Structure, which, at 35 457 CIDs implies over 10 000 more ligand structures than are indexed by PDB, for reasons that are not yet clear.

There seems to be no pattern to the discordances because each of the three has at least one example source where they are significantly the lowest. For the Human Metabolite Database (HMDB) it happens to be PC that is only 9 % of the In situ figures. In this case, dates indicate the last SID load into PC was November 2011, but CS has a more recent load from June 2017. For some reason the UC count from September 2017 is 2585 less. However, users need to know that HMDB underwent a major expansion in situ in October and so should search against this externally as the latest version.^15^ With comprehensive coverage in mind, users would thus need to check WikiPathways (unique to PC) as well as MetaboLights (unique to UC).^16^, ^17^ However, they would also need to check the first Recon set as loaded (also unique to UC) in October 2014. However, this has not been updated to the latest published Recon 2.3 set of 5324 metabolites.^18^ Thus, the important domain of metabolomics presents not only a mosaic of partial availability in different databases, but also needs the (hopefully pending) update of Recon (n.b., since Figure 3 was compiled, both HMBD and DrugBank have been updated in PC to 9765 and 114 297, respectively).

### 5.4 Vendors and virtuals

The major contributors to these databases by far are vendors. These offer the key advantage of enabling bioactivity research by the purchasing of structural analogous as an alternative, or complement, to de novo synthesis. The 293 vendor sources in CS cover 41.8 million compounds, reaching 67 % of total compounds. For PC the corresponding numbers are 284 sources merging to 63.0 million, coincidently also covering 67 %. We can determine that 29.5 million of the latter are unique structures. For UC there are also four vendors in the top‐ten sources (Table 2), but assessing the overall proportion is confounded by subsuming some of the same sources from PC. Notwithstanding the convenience of procurement, the opportunistic vendor “push” to nearly 70 % in both PC and CS (with ≈50 % of these as unique structures in the former), while commercially understandable, can be seen as a mixed blessing from several viewpoints. Firstly, content overlap between vendors becomes higher than users probably want (e.g., PC indexes 92 vendors for aspirin, including 12 Sigma catalogue SIDs for identical structures). Secondly, high novelty levels have to be caveated with doubts over structural quality because many of these turn out to be related to known CIDs via “same connectivity” matches in PC (e.g., where the vendor may not resolve the stereoisomers).

These issues can be inferred from inspecting Table 4 which shows a confusing pattern for just five vendor examples, including some websites not declaring exact totals. Loading dates are problematic, as can be seen in the case of eMolecules not updating since 2009 in CS or 2012 in UC but further confounded by deciding not to submit to PC at all. To add to the non‐obvious, it turns out that users can, in fact, find some eMolecules links within ZINC entries. The most striking numerical discordance is for Mcule with 5.6 million records in CS compared to 34 million in UC. While the former is an older load from October 2015 compared with the latter in February 2016, we can assume that the former are probably extant stock compounds (i.e., in pots) with the latter being predominantly virtual representations that have never been made. These are sometimes termed “make on demand” (MODs), where they have been enumerated with synthetic tractability in mind and the consequent likelihood of order fulfilment.

The statistics computed within UC support the inference of virtuals by showing that no less than 28 million of the Mcule submissions are unique (i.e., not in PC either); indeed, this is the single largest contributor to the increased size of UC over PC. The chequered history of some vendors was made manifest when PubChem removed Angene as a source in 2015. This was because they had reached 40 million CIDs by a combination of piggy‐backing (i.e., re‐submitting existing structures as their own SIDs, as in the NextBio case) and virtual enumerations (as evidenced when PubChem shrank by 8 million after their removal).

## 6 Utility Tips

So far this review has been more about problems than solutions (hence the title “Caveat Usor”). However, it is hoped that by unpicking at least some of the associated details (but by no means all), users can become more aware of potential pitfalls. They can then apply these insights to make comparable judgments for these or other resources. This section adds a few tips, some of which include caveats, to aid users in utility judgments. The first important point to note is that these databases are dynamic and consequently the snapshot of the four sets of numbers presented here may change fairly quickly (e.g., on the order of months). Consequently, checking current loading dates for sources of particular interest thus becomes important.

The decision of which of the three to search first (and the need to move on to either of the other two or not) clearly depends on the question being addressed. However, in the context of drug discovery (as the theme of this issue) the unequivocal first choice is PC. This is not only because of the unparalleled connectivity between Compound, Substance, BioAssay, PubMed, and Entrez but also the combination of filtering, mining, and analysis features. Selecting and intersecting are particularly powerful features of PC; for example, the search (“IUPHAR/BPS Guide to PHARMACOLOGY”[SourceName]) AND (“DrugCentral”[SourceName]) AND (“Therapeutic Target Database (TTD)”[SourceName]) AND (“DrugBank”[SourceName]) AND (“ChEMBL”[SourceName]) produced the result of 1110 CIDs in common between all five curated databases as a usefully cross‐corroborated drug set, where we can directly select the 374 that are in PDB (n.b., this takes only minutes on the advanced menu using the “Add to history” option, but is much slower executing the searches in the interface). In this way users can isolate essentially any subset. Another feature of PC that may be less well known is the ability to perform quality assessment in situ. The appropriate selection options are in the limits drop‐down menu under “Stereochemistry”. There are six settings in each case that enable the counting (and filtration) of both chiral and *E*/*Z* centers at different stringencies. This does not fix quality issues in various sources (as discussed by Lipinski et al.^6^), but does enable some extent of amelioration. Note also the ability to salt‐strip via the “Chemical Properties” menu with the “CovalentUnitCount” is another useful clean up step for inter‐source analysis or filtering sets for download.

Another tip that users may be less aware of is more of a caveat but is important to appreciate for interpretation of analysis results. This can be termed circularity as identical content between sources, for legitimate reasons (as opposed to piggy‐backing by straight copying). The issue has already been raised in the context of UC content, where several sources come in twice as independent loads and via PubChem subsumation. For PC it can be illustrated for the two important activity data submitters of ChEMBL and BindingDB. From the 1.7 million compound records in the former, 0.35 million are imported from PubChem confirmed BioAssays as well as 69 000 from BindingDB including 11 000 with curated patent activity data. Having introduced this reciprocity for content enhancement, BindingDB also import compounds with protein target mapped activity data from ChEMBL. Now both these sources submit to PC which means not only that some BioAssay data are therefore going in twice via ChEMBL, but the valuable BindingDB patent curation data also enter twice from both sources. These reciprocity arrangements are not hidden, but can easily confound users who might assume their respective content is independently acquired.

If users are designing new chemical structures they need to address the basic question “is there anything out there similar to what I am working on” not only as a basic cheminformatics question, but also for freedom to operate checks against patent sources. In such cases it would be prudent to check the other two databases. While UC is out in font for raw numbers, we can still only approach the question with exact match rather than similarity searches (although this may change). As the smallest member of the triumvirate, CS nonetheless also has unique content, possibly running into millions, but this remains undeclared.

**Figure 9:**
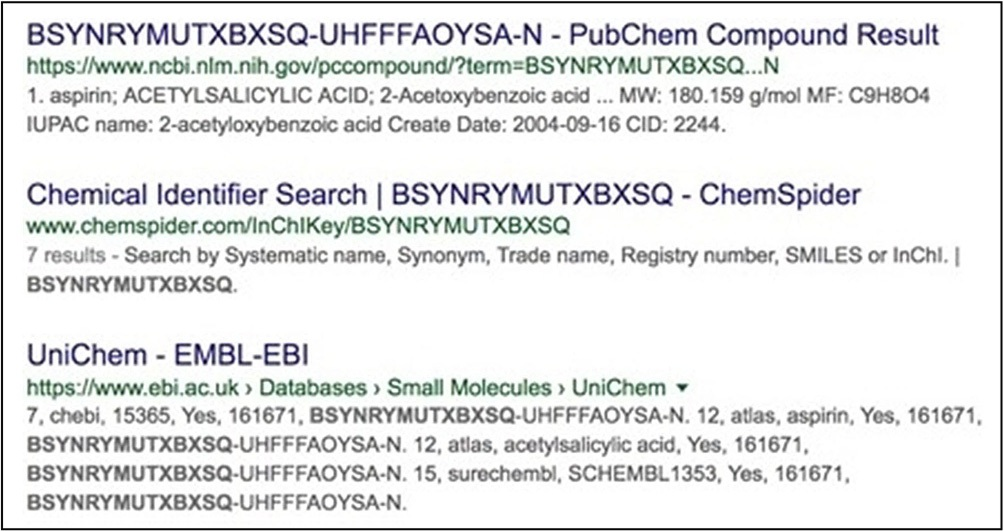
Google search with BSYNRYMUTXBXSQ (January 2018). This returned 858 matches in 0.29 seconds, from which the top‐three are shown.

For novelty checking there already exists a crude approximation to the merging of all three databases in the form of searchable IKs as indexed in Google.^21^ This can demonstrated by a simple search with the connectivity layer inner Key for aspirin (since Ref. 21 was published in 2013, it turns out that full IK searches have become more susceptible to various kinds of false positives). The results are shown in Figure 9.

In this case, we see that each of the three databases have in fact emerged as the top matches, but many other database links are in the hit list, which is remarkably clean in including very few false positives (note also the searches execute faster than internal searches within the sources).

## 7 Conclusions

The resources reviewed herein indicate that, on the one hand, researchers exploring bioactivity space and the wider chemical neighborhood for drug discovery have never had it so good. On the other, they are confronted with complex differences, including non‐obvious ones, between these resources that have a direct bearing on utility. In this regard, a key issue needs to be highlighted in an attempt to understand the “why” of divergence. While the “who‐has‐what” questions can be laboriously addressed by comparisons of the type done in this work, such results do not explain the causes of this divergence, even where resources are described in detailed publications as well as internal documentation.

Notwithstanding, implicit divergence trends emerge from this work. For PC this includes the unique breadth of connectivity and the embracing of patent extraction sources to the level of 22 million CIDs.^9^ For CS the alternative choice has been made of eschewing patent chemistry in the interests of overall structural quality (mainly due to the tendency of automated extraction to convert fragmented IUPAC names). The crowd sourcing element is also designed to enhance quality in CS (although the statistics of entry names and/or structure corrections as a consequence of this have not been declared). For UC the focus on EBI databases is clear, but in addition, by subsuming structures from external sources, they have managed to not only overtake PC by nearly 60 million but also SciFinder by 20 million.

However, both from discussions with individuals and observing changes over the years, an undeclared diverging influence emerges. This is that, understandably, database teams have their work cut out simply to maintain the status quo while also pursuing both content and feature expansion. This leaves little reserve capacity for longer‐term strategic changes in direction necessary to significantly shift balances of content. This could include, for example, encouraging submissions of novel chemical space, expanded activity data sets, deprecating sources where value has declined, filtering patent extractions at higher stringencies, and resisting vendor pushes of highly overlapping or virtual content. We therefore need to accept (but not see as a criticism) a certain ad hoc element to resource divergence, while noting at the same time the positive consequence of complementarity.

In terms of cumulative coverage of all three databases there is also an important caveat in the increasing numbers of “boutique” standalone databases with valuable internal small‐molecule indexing related to different types of bioactive chemistry. Some of these are either not subsumed into the sources above (i.e., have decided not to submit) or have been languishing many years out of date. Inspection of the 2018 Nucleic Acids Research Database issue indicates examples of both the former (e.g., SuperDrug2) and the latter (e.g., Therapeutic Target Database had not updated in PC since 2012 but eventually refreshed in December 2017 with a major expansion to 22 134).^19^, ^20^ So wouldn't it be great if there was just a one‐stop portal where: a) all standalone databases with significant value committed to submit to, b) they ensured their pulls or pushes were synchronized with their internal releases, and c) they agreed to harmonize their different structure standardization processes? This would certainly be high on everyone's wish list looking at these major databases from the outside. However, given the different funding models, chemistry rules, and stakeholder interests on the inside, this does not seem so likely in the near future, but we can always hope.

*Note added in proof: in the months elapsed between submission and proofing there have been changes in specific numbers (e.g., CS reducing their sources). It was not possible to pick up all of these instances, but they do not alter the general points*.

## Conflict of interest

*The author declares no conflict of interest*.

## Biographical Information

Dr. Christopher Southan has been a Senior Cheminformatician for the IUPHAR/BPS Guide to PHARMACOLOGY at the University of Edinburgh since 2013, but works remotely from Sweden. Prior to this he set up TW2Informatics, working on patent informatics for SureChem (2011/12) and AstraZeneca (AZ) on Chemistry Connect and Pharma Connect (2009–2011). In 2008/9 he coordinated the ELIXIR Database Provider Survey at EBI and was Team Leader in AZ Molecular Sciences (2004–2007), preceded by senior bioinformatics positions at Oxford Glycosciences, Gemini Genomics and SmithKline Beecham. His PubMed papers (Southan C) encompass pharmacology, drug discovery, bioactivity database comparisons, bioinformatics, and cheminformatics. Further information is on his LinkedIn profile, tweets at @cdsouthan and blogs at https://cdsouthan.blogspot.se/.